# Revisiting the Governance–Dividend Nexus: The Mediating Role of Corporate Social Responsibility

**DOI:** 10.12688/f1000research.167892.1

**Published:** 2025-09-09

**Authors:** Riffat Shaheen, Hussaini Bala, Ahmad Haruna Abubakar, Supriya Lamba Sahdev, Armaya'u Alhaji Sani, Ghousia Khatoon, Umair Zahid

**Affiliations:** 1 1) Department of Management Sciences, University of Wah, Wah Cantt., Punjab, Pakistan; 2 Accounting Department, Tishk International University, Erbil, Iraq; 3 Management and Science University Faculty of Business Management and Professional Studies, Shah Alam, Selangor, Malaysia; 4 Alliance School of Business, Alliance University, Bengaluru, Karnataka, India; 5 Department of Business Management, Global Banking School, University of Suffolk, London, England, UK

**Keywords:** Corporate governance, Corporate social responsibility, Dividend policy, China

## Abstract

**Background:**

Despite plentiful research on the link between corporate governance (CG) and dividend policy, the mediating impact of corporate social responsibility (CSR) on the CG –dividend policy link remains unexplored. This study investigates the mediating impact of CSR on the CG –dividend policy link, and explores the mechanism through which CSR mediates this relationship.

**Methods:**

Firms listed on the Shanghai and Shenzhen stock exchanges are considered as a study sample. The data collection period ranged from 2012 to 2021. The final sample included 15,800 firm-year observations.

**Results:**

The results findings indicate that firms with strong CG tend to pay low dividends, consistent with the substitution hypothesis, in which dividends and CG act as substitutes for each other. In addition to establishing a direct link between CG and dividend policy, our study significantly contributes to the extant literature by presenting both theoretical proposition and empirical evidence on the mediation effect of CSR in the above-mentioned relationship. We find that CSR mediates the corporate governance- dividend policy relationship, which implies that in comparison to corporate governance, CSR has a more dominating impact on firms’ dividend policy decisions, and better-governed firms are more likely to engage in CSR activities to protect their stakeholders; consequently, they prefer to hold or invest cash instead of paying dividends because CSR engagements lower the cost of equity capital. These findings were corroborated by a set of robustness tests.

**Conclusions:**

Our results have several implications for firms, regulators, and investors. Firms can use high dividend payouts to compensate for poor investor protection and to maintain good relationships with investors. When making investment decisions, investors are advised to consider socially responsible firms because of their strong CG structure. Finally, policy makers should give special consideration to CSR in order to reduce environmental and social problems and to enhance the related standards to ensure the safety and security of all stakeholders and hence reduce global accusation and pressure.

## 1. Introduction

It is widely acknowledged that in recent years, different corporate scandals (e.g., WorldCom, Parmalat, Xerox, and Enron) and financial crisis outbreaks are likely to be caused by bad management practices. This situation highlights the significance of corporate governance (CG) practices; hence, CG has become a central concern in various discussions in the literature. Generally, CG provides several advantages to firms (e.g., reduced capital cost, strong corporate reputation, and improved performance) and stakeholders, including investors (e.g., legal protection and increased shareholder worth) and society (e.g., protection against corruption and an appropriate environment for investment), to enhance firms’ efficiency and competition and to facilitate capital market development. ^1, 2^ However, compared with developed economies, firms and investors in emerging economies are less likely to enjoy these benefits because of poor CG and low investor protection. The central concern of CG is ensuring that investors receive fair investment returns, whether in the form of dividends or capital gains. However, in situations where governance is inadequate, investors may be threatened with expropriation by management or controlling shareholders if free cash flows are misused. ^3, 4^ Because of expropriation concerns, investors are likely to choose dividends above capital gains.

Corporate governance (CG) is an important factor in dividend policy ^5, 6^ and dividend payout decisions are regarded as essential components of company policy. Finance researchers find dividend policy one of the most fascinating and contentious research topics because of its enigmatic character and significance in a company’s CG. The dividend policy and CG link have been studied in both developed and emerging economies; however, earlier researchers mostly focused on this link in the context of developed economies. ^7^ Since emerging countries’ financial markets are found to have a substantial influence on global financial activity and are receiving a large portion of world equity investments, investors have begun to take more interest in emerging markets’ dividend policy choices. Our investigation of dividend policy and CG links is based on China’s world’s largest emerging economy. The rising interest of investors in dividend payouts of Chinese listed firms is due to several unique features of the country. For example, China enjoys the position of the largest emerging economy in the world and the second largest economy overall. In publicly quoted Chinese corporations, dividend policy is the core financial decision ^8^ because dividend payments generated approximately half of the MSCI China Index’s total returns from 1999 to 2012 [ 1]. Further, in contrast to developed economies, investors in China are more dependent on dividends to obtain additional information because stock repurchases are strictly constrained by Chinese company law, and firms are hardly allowed to repurchase their stocks. ^9^ Finally, due to poor investor protection and poor law implementation, Chinese firms experience large variations in dividend policies compared with their Western counterparts ^10– 12^ who enjoy stable dividends.

Despite being the fastest growing economy, Chinese firms are found to have severe agency problems because most of them are owned by the Chinese government, which results in high agency costs. ^13^ Moreover, shareholder protection rights and governance practices in emerging economies such as China are not strong enough to protect minority shareholder rights. CG and corporate social responsibility (CSR) are regarded as two sides of the same coin. The focus of both CG and CSR is on ethical practices in an organization, and they help firms respond effectively to their stakeholders and the environment in which they operate. ^14^ Further, dividend policy is an important tool firms can use to reduce conflicts of interest and satisfy their shareholders. ^7, 15– 17^ Consequently, it is expected that better CG and CSR involvement can help firms protect their shareholders from expropriation threats through dividend payments, because dividends can be used to provide more benefits to investors and hence protect them against expropriation. ^6, 18^

Given China’s impressive growth over the past couple of decades, the CSR of Chinese firms has received much attention from researchers. In contrast to developed countries where CSR is used as a means to show the company as a legal social entity to its stakeholders, Chinese firms consider CSR as a business tactic for gaining and maintaining market share. ^19, 20^ In addition, these firms are involved in CSR activities to enhance their image, consequently improving shareholders’ financial performance and wealth of shareholders. ^21^ China’s rapid growth has caused severe damage to the environment and society. Examples include violation of food safety laws, heavy use of chemical fertilizers, excessive use, and endorsement of harmful drugs. Recently, China has made rapid assessments of CSR ^22^ and introduced several CSR reforms; however, Chinese firms’ CSR practices are still in an early stage, and firms are under greater pressure to be involved in CSR-related activities. ^23^

Despite plentiful research on the link between corporate governance (CG) and dividend policy, the mediating impact of CSR on the CG–dividend policy link remains unexplored. Considering the importance of dividend policy, corporate governance, and CSR performance in the capital markets of China, the central focus of this study is to explore the mechanism through which a firm’s CG influences its dividend policy in the presence of CSR, which is the main contribution of this study. Many researchers have attempted to study the link between CG and dividend policy. However, to the best of our knowledge, previous literature lacks the consideration of three variables together, and none of the studies have investigated this link from the perspective of firms’ engagement in CSR activities. The distinctive aspect of our research is that we explore the CG – dividend policy causal link from the perspective of the mediating role of firms’ CSR performance to identify whether a firm’s engagement in CSR activities influences this causal relation, and compared to corporate governance, CSR has a more dominant impact on dividend policy. Therefore, our study provides pioneering evidence on the mediating role of CSR in the impact of CG on dividend policies and is expected to make a significant contribution to the existing literature. Moreover, findings on the link between CG and dividend policies are mixed in nature. Our study contributes to the literature on CG - dividend policy links, supporting the substitution hypothesis. In addition, most previous studies in China have investigated the individual impacts of different CG mechanisms on dividend policy. By contrast, we used a comprehensive CG index covering the most important aspects, including BODs, disclosure and transparency, audit committees, remuneration committees, and shareholder rights.

Our dataset consisted of 15,799 firm-year observations from 2012 to 2021. Using Baron and Kenny’s (1986) mediation approach, we find evidence in favor of our main hypothesis that CSR mediates the causal relationship between corporate and dividend policy. The findings support our second argument about the mediating role of CSR: better CG leads to high CSR involvement, which in turn reduces dividend payments because of a firm’s preference to hoard or invest cash instead of paying dividends. Our first argument, that better CG can result in high CSR involvement, which in turn leads to high dividend payments through increased reputation and earnings of the firm, is not supported by the results.

The remainder of this paper is organized as follows. Section 2 reviews the existing literature and develops the hypotheses. Section 3 explains the sample selection criteria, data sources, variables, measurements, and methodology used. Section 4 presents and discusses the empirical analysis results. Section 5 concludes the paper and Section 6 provides the implications of the study.

## 2. Review of Literature and Hypothesis Development

### 2.1 Corporate Governance and Dividend Policy

Apart from other corporate decisions, shareholders are primarily concerned with dividend policy decisions, which are a central concern in firms’ financial decisions of the firms. ^24^ It is evident from the existing literature that, in firms’ corporate governance, dividend policy has received greater importance as an influential key ^25^ and in Chinese initial public offerings (IPOs), CG is an important determinant of dividend policy. ^26, 27^ From the agency theory perspective, in firms with higher ownership concentration, minority shareholders are expected to be at expropriation risk when large shareholders, by using their voting rights, tend to affect the firm’s policies and decision-making for their private benefits. The threats of expropriation are more common in environments (e.g., emerging economies) with weak CG and low investor protection according to Ref. 3. Moreover, large shareholders prefer to generate their own benefits when they gain full control of the firm and do not share these benefits with minority shareholders. ^28^ In East Asian and European economies, prominent agency problems arise due to minority shareholders’ expropriation by controlling shareholders. ^29^ The dividend policy is significantly determined by agency costs and can be used to limit minority shareholders’ expropriations by reducing corporate wealth under insiders’ control. ^30, 31^

The ultimate goal of CG is to ensure that investors who supply finances to firms receive their investment returns is the ultimate goal of CG. ^28^ Shareholders receive return on investment either in the form of capital gain or dividends however, they prefer dividends to overcome the expropriation fear from controlling shareholders, as reported by Ref. 32 that, “dividends (a bird in hand) are better than retained earnings (a bird in the bush) because the latter might never materialize as future dividends.” Our discussion of the link between CG and dividend policy is based on the two most widely used agency models of dividends proposed by Ref. 32. The first model known as “the outcome model” of agency theory suggests that to restrict managers’ private benefits of free cash flows, firms with strong CG tend to pay higher dividends. In other words, shareholders have the power to influence dividend policies when they have greater rights through strong CG or legal protection. ^10^ Hence, better governance helps firms reduce agency conflicts through higher dividends, meet shareholder demands, and enhance their trust in management, and consequently, the firm. The outcome model predicts a positive association between dividend policies and CG quality. Many studies have supported this model. For example, using a sample of Canadian firms, Ref. 33 showed that dividend yield is positively associated with board composition and governance quality. Reference 34 report that in the case of US and Indian companies, dividend payouts are positively associated with governance quality. They analyzed the interrelation between shareholder protection at both firm and country levels. Reference 35 claims high payouts in strong CG firms for a sample based on emerging markets. Based on survey data from listed firms in Korea, a positive association is found between good CG and dividend payouts. ^36^ Reference 32 find support for the outcome model by using a sample of 33 countries and report that in countries with strong investor protection, firms have a higher dividend payout ratio than countries with poor investor protection. Dividend payouts are higher and more consistent in firms where CG is stronger. ^25, 37^ Reference 38 assert that firms with stronger shareholder rights pay more dividends because dividend policy is expected to be the outcome of the governance regime. Reference 39 found a positive association between dividend payouts and strong shareholders in Dutch firms. In countries with stronger creditors’ rights, firms tend to have higher payouts ^40^ supporting the outcome model. Recently, Ref. 41 studied the influence of family engagement and CG on dividend policies. They explore non-financial companies in Morocco and reveal that GC mechanisms have a strong influence on divided policy in Morocco.

Conversely, the substitution hypothesis of agency theory proposes that, to have capital market excess and maintain good shareholder relations, higher dividend payouts can be used as a substitute for weak governance. ^42, 43^ In firms with weak shareholder rights, building reputation by establishing good relationships with investors is a key concern, and dividend payouts play a vital role in building this reputation. By contrast, reputation and dividend payouts are less needed in firms with strong corporate governance. ^44^ Therefore, firms with weak CG should have large dividend payouts to compensate for their weak shareholder rights. Since dividend payouts help to reduce managerial expropriations through a decrease in free cash flows, dividend payouts and CG are expected to have an inverse relationship ^38^ and Under weak governance, dividends act as substitutes for poor investor protection. Many studies have demonstrated the substitution effect of dividends. For example, CG strength is inversely associated with dividend payouts. ^38, 45– 48^ The strength of corporate governance is measured by using CG index of Gompers (2003). ^49^ Reference 50 analyzed a sample of master limited partnerships and reported that low-quality governance firms make high dividend payments; however, these payments are value-destroying, as they negatively affect firms’ cash holdings and firm value. CG can be effectively substituted by dividends to reduce agency costs. ^48^ Reference 51 found a significant negative association between dividend policy and CG mechanisms, such as being familiar with auditors, timeliness, and shareholder rights. Managerial entrenchment is positively associated with both the level and propensity for dividend payouts. ^52^ Using the governance index of Gompers ^49^ along with institutional block holdings and board structure, Ref. 45 report a negative relationship between dividend payouts and governance quality in favor of the substitution effect. In contrast to low-quality governance firms, firms with high quality governance predicted as dividend payers tend to make fewer dividend payments. ^47, 53^ He further argued that, in firms with weak governance, dividend initiation receives a positive response from shareholders, indicating the substitution of dividends for weak governance.

In Chinese firms, prominent conflicts of interest exist between minority shareholders and large dominant shareholders, and there are more expropriation opportunities for majority shareholders due to weak investor protection and creditor rights. ^6, 54^ Minority shareholders are more likely to suffer from self-serving behavior, such as excessive compensation, expropriation, and tunneling. Because Chinese firms suffer from weak shareholder protection and poor CG (e.g., minority-majority shareholder conflicts and state-owned equity control), they tend to pay high dividends as a governance control mechanism. ^4, 6^ In other words, Chinese firms are likely to use dividends as substitutes for poor governance to reduce conflicts between minority and majority shareholders. Considering Chinese firms as a sample for our research study, we develop the following hypothesis:

H1:
*Firms with strong (weak) corporate governance tend to pay low (high) dividends.*

### 2.2 CSR and Dividend Policy

The growing interest in corporate social responsibility and increased importance of dividend policy in corporate finance literature based on its “puzzle” like nature have attracted researchers’ attention to explore the nature of link between them and the theories underlying this relationship. However, most of the literature (e.g., Refs. 14, 55, 56, 57) focuses on developed economies, and only a few studies have explored this relationship in emerging economies, such as China and few others (eg., Refs. 58, 59). According to Ref. 56, the link between CSR and dividend policy is guided through two channels: the earnings channel and equity cost of the capital channel. Our hypotheses on the CSR dividend policy link are based on these two CSR views on dividends.

According to the cost of equity capital channels, instead of paying dividends, firms tend to hold or invest cash because their involvement in CSR activities can reduce the equity cost of capital through its risk premium. ^56, 60^ A firm’s CSR practices lower its financial constraints either by reducing the cost of bank loans or the cost of capital. ^61, 62^ High CSR reduces equity capital costs by lowering the associated risk premium because socially responsible firms tend to enjoy good reputations, ^63, 64^ have good management practices and low information asymmetry, ^57, 65^ align interests between shareholders and other stakeholders, ^66^ loyal customers or investors ^67, 68^ and have low risk and good governance. ^69^ Since the opportunity cost of holding cash is low (high) in the case of a low (high) equity cost of capital, firms’ incentives to hold cash increase (decrease). ^70^ On the other hand, firms might have more (less) incentives to use cash for investment purposes because financial constraints are less (more) strict for firms in the case of lower (higher) equity cost of capital. ^56, 58, 71^ Moreover, being financially less constrained, CSR firms can invest in projects that appear unprofitable and unfeasible because of high capital costs and low availability of external finance. These arguments suggest that CSR involvement reduces the equity cost of capital because firms are more likely to hoard or invest cash instead of paying dividends. In other words, the equity cost of the capital view of CSR and dividend policy links predicts a negative association between CSR and dividend policy.

In contrast to the first CSR view of dividends, the earnings channel view argues that CSR affects dividend policies through its effect on earnings. Earnings have been identified as a crucial determinant of dividend policy and firms with greater earnings ability are more likely to pay dividends. ^72, 73^ Socially responsible firms tend to have enhanced earnings, which leads to higher dividend payments. The mechanisms through which CSR-related activities enhance firms’ earnings include efficient management and good relationships with stakeholders. ^74– 76^ A better firms-stakeholder connection helps in developing competitive advantage, ^77, 78^ reducing transaction costs, ^79^ cash flow shocks in the occurrence of some negative events ^80^ and lower firm risk. ^56^ In addition, CSR activities are associated with higher ethical standards and greater transparency, ^81– 83^ enhanced corporate reputation, ^84^ greater customer loyalty, high commitment of employees, a high degree of suppliers’ cooperation ^85^ and a reduced annual labor turnover rate. ^86, 87^ These factors significantly contribute to the earnings-generating ability of firms with strong involvement in CSR activities, which, in turn, increases their dividend payouts and enables them to pay dividends (if non-payers). Based on the preceding arguments, firms’ involvement in CSR activities can result in dividend payouts through increased earnings, as CSR activities are considered positive net present value projects. Hence, stronger CSR involvement puts firms in a better position to pay dividends and to pay higher dividends. The earnings view predicts a positive association between CSR and dividend policy. Reference 88 examined CSR and dividend policies in India. They revealed that CSR has a constructive influence on dividend disbursements. In addition, ^89^ explore how CG influences the link between information asymmetry and dividend disbursement in an emerging economy. They found that dividend payouts are inversely inclined by information asymmetry issues. They also find that the link between information asymmetry and dividend strategy is less prominent in companies with robust CG systems. This finding is in line with the argument that companies encounter lower agency and asymmetric information issues when they pay higher dividends.

From the perspective of Chinese firms, evidence on the link between CSR and dividend payouts remains ambiguous. For example, Ref. 58 provide evidence in favor of earnings channels, while Ref. 12 support the equity cost of capital channels. Therefore, based on the two divergent views on the link between CSR and dividends, we develop the following hypotheses:

H2a:
*Firms with involvements in CSR activities tend to pay low dividends (equity cost of capital channel).*

H2b:
*Firms with involvements in CSR activities tend to pay high dividends (earnings Channel).*

### 2.3 The mediating role of CSR

As the function of CSR is to make firms accountable to their stakeholders, firms committed to CSR practices can identify and assess the demands of different stakeholders. These CSR commitments facilitate a strong link between a firm and its stakeholders by considering their needs and demands. According to Ref. 90, CG and CSR are interdependent, and CSR as a firm’s obligation to society can be regarded as an external mechanism of corporate governance. The main argument in this paper is that CG is likely to influence the corporate social responsibility (negatively/positively), which in turn influences dividend payments. Our expectation of CSR’s mediating role in the link between CG and dividend policy is based on two arguments. First, in the context of stakeholder theory, firms with effective CG invest more in CSR to ensure the protection of all stakeholders beyond shareholders. ^91, 92^ Further, the earnings channel predicts that firms with stronger CSR involvement are expected to pay higher dividends. ^83, 93^ Based on these theories, it is argued that better CG can result in high CSR involvement, which in turn leads to high dividend payments through the increased reputation and earnings of the firm. Therefore, CSR is expected to facilitate a link between CG and dividend policies.

Second, stakeholder theory suggests that better-governed firms provide interest protection to all stakeholders beyond shareholders by investing more in CSR. In addition, the cost of equity channels suggests that firms with stronger CSR involvement tend to pay low dividends, as in this case, firms prefer to hoard or invest cash instead of paying dividends due to the low cost of equity. ^56, 94^ Accordingly, it is argued that better CG can result in high CSR involvement, which, in turn, reduces dividend payments because of a firm’s preference to hoard cash or invest it instead of dividend payments. In this context, we expect CSR to mediate the link between CG and dividend policy. The following hypothesis was developed to test our arguments regarding mediation:

H3:
*CSR mediates the link between corporate governance and dividend policy.*

### 2.4 Conceptual framework

**Figure 1.**
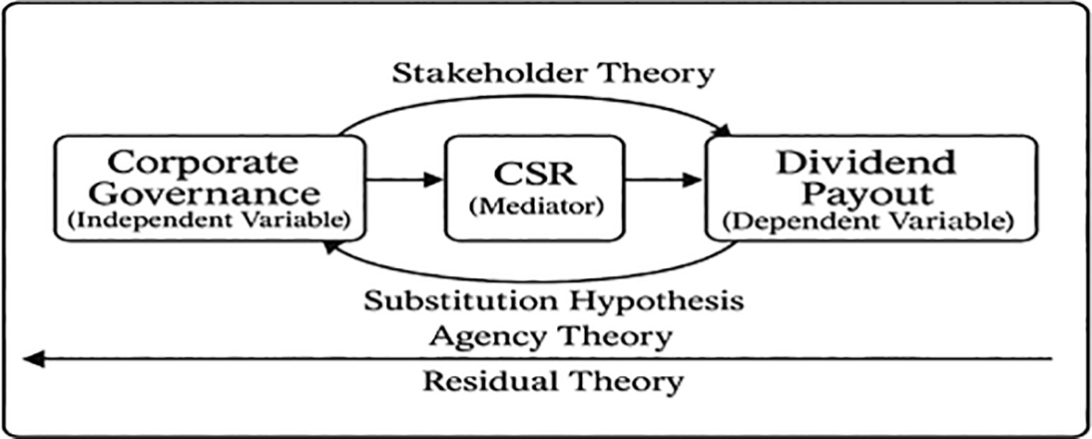
CSR mediating influence of corporate governance in the governance-dividend relationship. This figure illustrates the mediation analysis results, showing the direct and indirect effects of governance mechanisms on dividend payout through CSR engagement. Path coefficients are reported with significance levels.

Based on the arguments above, the conceptual framework of mediation to explore the link between CG and dividend policy with the mediating impact of CSR is shown in Figure 1.

## 3. Methodology

### 3.1 Sample description

Firms listed on the Shanghai and Shenzhen stock exchanges are considered as a study sample to investigate the link between CG and dividend policy with the mediating role of CSR. The sample is selected using the 2012 China Securities Regulatory Commission (CSRC) industry classification, under which the firms are categorized into 19 different industrial sectors. The data collection period ranged from 2012 to 2021 based on data availability and an attempt to use the most recent data as much as possible. The final sample included 15,800 firm-year observations.

### 3.2 Data sources

CSR data were collected from the Hexun database, which offers the CSR evaluation score of listed Chinese firms beginning in 2010. Many previous studies have used CSR data from this database. ^58, 95– 98^ To appraise the corporate social responsibility performance of Chinese firms, Hexun.com ranks first, and provides a good and comprehensive source of CSR data. ^58, 96^ Moreover, it comprehensively covers all critical stakeholder groups, including shareholders, employees, suppliers, customers, environment, and community in its assessment framework. ^96^ The data on all other variables, including CG and control variables, were collected from the China Stock Market and Accounting Research (CSMAR) database.

### 3.3 Research variables

The dependent variable is the dividend policy, for which the dividend payout ratio (DPR) is used as a proxy. Many researchers have used the dividend payout ratio as a proxy for dividend policy for example, Refs. 36, 56, 58 and 99– 102. The dividend payout ratio (DPR) was calculated as dividends per share divided by earnings per share. We use this measure because, according to Refs. 24 and 93, it establishes a direct connection between distribution and earnings.

The independent variable corporate governance was measured using the corporate governance index (CGI). CG is considered a multidimensional notion, defined as the mechanism used for the protection of different stakeholders’ interests. ^103^ Many cultural, economic, environmental, legal, historical, and political factors affect corporate governance. Following Munisi and Randøy, ^104^ we used a comprehensive index of CG covering important aspects related to its different dimensions, including BODs, disclosure and transparency, audit committees, remuneration committees, and shareholder rights, as recommended in different CG codes and used in previous literature. To calculate the aggregate CGI, we first calculated the sub-index for each dimension by assigning a value of one if the statement was true and zero if the statement was not true. The aggregate CGI was then calculated by adding all values. All sub-indices were given equal weights. The composition of the CG index is presented in Table 1.

**Table 1.** The Composition of Corporate Governance Index (CGI).

No.	CG Indicators	If statement is true/not
**1.**	**BODs (Sub Index I)**	
a.	CEO duality exists; CEO is also the board’s Chairperson	1/0
b.	Board’s chairperson is non-executive director	1/0
c.	Company specifies the directors’ categories	1/0
d.	At least 2/3 of the board is consist of non-executive directors	1/0
e.	Company shows the number of board meetings held	1/0
f.	Board has a committee for CG	1/0
g.	Board has a nomination committee	1/0
**2.**	**The Audit Committee (Sub Index II)**	
a.	Company has an audit committee	1/0
b.	The audit committee’ s chairperson is a non- executive director	1/0
c.	All committee members are non-executive directors	1/0
d.	Board’s chairperson and the audit committee chairman/its members are different individuals	1/0
e.	Company shows the number of committee meetings held	1/0
**3.**	**Transparency & Disclosure (Sub Index III)**	
a.	Company has a remuneration committee	1/0
b.	The remuneration committee’s chairperson is a non-executive director	1/0
c.	All committee members are non-executive directors	1/0
d.	Company shows the number of committee meetings held	1/0
**4.**	**The Remuneration Committee (Sub Index IV)**	
a.	The composition of remuneration committee is disclosed by the company	1/0
b.	The composition of audit committee is disclosed by the company	1/0
c	Each director’ total remuneration is disclosed by the company	1/0
d.	The CEO’ remuneration is disclosed by the company	1/0
e.	The senior officers’ professional/work qualification is disclosed by the company	1/0
f.	The senior officers’ academic qualification is disclosed by the company	1/0
g.	The senior management team’s remuneration is disclosed by the company	1/0
h.	The directors’ professional/work qualification is disclosed by the company	1/0
i.	The directors’ academic qualification is disclosed by the company	1/0
j.	The ages of directors are disclosed by the company	1/0
k.	Each director’s appointment date is disclosed by the company	1/0
l.	The external auditor of the company is “big 4” audit firm	1/0
m.	The details of share ownership are disclosed by the company	1/0
n.	Company displays information on performance of stock market & stock prices	1/0
o.	Company shows remarks on its financial outcome	1/0
p.	Company provides information on its activities concerning social responsibility	1/0
**5.**	**Rights of Shareholders (Sub Index V)**	
a.	Company exercises the principle of one share -one vote	1/0
b.	All directors are elected every year	1/0
c.	Company shows that proxy voting is allowed	1/0

Corporate Social Responsibilty (CSR) is used as a mediator. This is calculated using firms’ CSR scores from the Hexun database. This database calculates the CSR scores of Chinese listed firms based on five dimensions: employees, environment, community, supplier and customer, and investors. Each of these dimensions further consists of different sub-dimensions and is given different weights depending on the industries to which the firm belongs. Consistent with previous studies (eg. Refs. 58, 96, 98, 105), we calculated CSR scores as a total of four sub-social responsibility scores: employees, environment, community, supplier, and customer. First, the individual score for each sub-dimension was calculated, and then the aggregate score was calculated as the average of the sub-dimensions’ individual scores.

In addition, we control for previously identified factors that can affect dividend policy. To consider the firm’s financial condition, we include firm size, leverage, market-to-book ratio, profitability, ratio of retained earnings to total assets, and research and development expenditure as control variables. Firm size (SZ) is expected to affect both firms’ debt and dividend policies. ^106^ Larger firms are more diversified, have less volatility and more regular cash flows, and can maintain debt at high levels, as they are less likely to go bankrupt ^107^ and large firms with good financial conditions usually pay dividends. ^108^ Because leveraged firms are riskier in their cash flows and try to avoid external capital costs ^16, 109^ therefore firms’ dividend behavior is also expected to be affected by leverage. According to Ref. 110, through the market to book ratio (MTB), firms convey information to shareholders about their smooth dividend payments; hence, MTB is also added as a control variable. Moreover, profitability (ROA) is also considered an important determinant of dividend policy in the literature and is expected to influence dividend behavior because it reflects a firm’s resource availability after investments are funded. ^6, 111^ R& D expenditure is used as a proxy for firm growth opportunity and is expected to influence dividend policy because as compared to non-growth firms, growth firms pay fewer dividends. ^112, 113^ Further, the ratio of retained earnings to total assets (RETA) significantly determines dividend payouts, and firms with a higher proportion of earned capital instead of contributed capital tend to pay more dividends; therefore, Ref. 114 added RETA as a control variable in the model. A summary of the variables, their calculations, and data sources is provided in Table 2.

**Table 2.** Variables’ Measurements and Data Sources.

Variables	Abbre.	Calculation	Reference	Data source
Dividend payout	DPR	Dividend per share/earnings per share	^6, 115^	CSMAR database
Dividend dummy	DD	DD=1 if firm is dividend payer, 0 otherwise.	^38, 116^	Authors own calculation
Corporate Governance Index Corporate Social Responsibility Firm Size	CGI CSR SZ	Aggregate score of five sub-indices of CG including BODs, disclosure& transparency, audit committee, remuneration committee, and shareholder rights Aggregate score of CSR dimensions including community, environment, customer& supplier, and employee Natural log of total assets	^104^ ^96, 58, 98^ ^6, 100, 116^	Authors own calculation. Information on CG indicators is collected from CSMAR database including annual reports of firms Hexun database CSMAR database
Return on Assets	ROA	EBIT/total assets	^115, 50, 116^	CSMAR database
Market to Book Ratio	MTB	Market Value/book value of common stocks	^36, 98^	CSMAR database
Leverage	LEV	Total debts/total assets	^38, 36, 58, 50^	CSMAR database
Retained Earnings to Total Assets	RETA	Retained earnings/total assets	^107, 117^	CSMAR database
Research and Development expenditures	R&D	R&D expenditures/total assets	^113^	CSMAR database

### 3.3 Estimation model

To investigate the mediating impact of CSR on the causal link between CG and dividend policy, we used a fixed-effects regression model identified through panel data model identification tests. Our analysis is based on the mediation approach suggested by Baron and Kenny, ^118^ which consists of the following steps.

$${\mathrm{DPR}}_{\mathrm{it}}=\beta_{0}+\beta_{1}{\mathrm{CGI}}_{\mathrm{it}}+\beta_{2}{\mathrm{SZ}}_{\mathrm{it}}+\beta_{3}{\mathrm{ROA}}_{\mathrm{it}}+\beta_{4}{\mathrm{MTB}}_{\mathrm{it}}+\beta_{5}{\mathrm{LVG}}_{\mathrm{it}}+\beta_{6}{\text{RETA}}_{\mathrm{it}}+\beta_{7}R\&D_{\mathrm{it}}+\varepsilon_{\mathrm{it}}$$ (1)

$${\mathrm{CSR}}_{\mathrm{it}}=\beta_{0}+\beta_{1}{\mathrm{CGI}}_{\mathrm{it}}+\beta_{2}{\mathrm{SZ}}_{\mathrm{it}}+\beta_{3}{\mathrm{ROA}}_{\mathrm{it}}+\beta_{4}{\mathrm{MTB}}_{\mathrm{it}}+\beta_{5}{\mathrm{LVG}}_{\mathrm{it}}+\beta_{6}{\text{RETA}}_{\mathrm{it}}+\beta_{7}R\&D_{\mathrm{it}}+\varepsilon_{\mathrm{it}}$$ (2)

$${\mathrm{DPR}}_{\mathrm{it}}=\beta_{0}+\beta_{1}{\mathrm{CSR}}_{\mathrm{it}}+\beta_{2}{\mathrm{SZ}}_{\mathrm{it}}+\beta_{3}{\mathrm{ROA}}_{\mathrm{it}}+\beta_{4}{\mathrm{MTB}}_{\mathrm{it}}+\beta_{5}{\mathrm{LVG}}_{\mathrm{it}}+\beta_{6}{\text{RETA}}_{\mathrm{it}}+\beta_{7}R\&D_{\mathrm{it}}+\varepsilon_{\mathrm{it}}$$ (3)

$${\mathrm{DPR}}_{\mathrm{it}}=\beta_{0}+\beta_{1}{\mathrm{CGI}}_{\mathrm{it}}+\beta_{2}{\mathrm{CSR}}_{\mathrm{it}}+\beta_{3}{\mathrm{SZ}}_{\mathrm{it}}+\beta_{4}{\mathrm{ROA}}_{\mathrm{it}}+\beta_{5}{\mathrm{MTB}}_{\mathrm{it}}+\beta_{6}{\mathrm{LVG}}_{\mathrm{it}}+\beta_{7}{\text{RETA}}_{\mathrm{it}}+\beta_{8}R\&D_{\mathrm{it}}+\varepsilon_{\mathrm{it}}$$ (4)

First, the effect of the independent variable (CGI) was tested on the dependent variable (DPR). Here, the independent variable was expected to show a significant relationship with the dependent variable. In the second step, the link between the mediation variable (CSR) and the independent variable is tested, and the independent variable is expected to have a significant influence on the mediator. In the third step, the effect of the mediation variable on the dependent variable was tested, and the results needed to be significant. In the final step, both the independent variable and mediator were tested on the dependent variable. Here, the effect of the independent variable (beta coefficient of CGI in the final regression model) on the dependent variable is expected to be less in absolute terms compared to the effect in the first regression model. ^1^

## 4. Empirical Results and Discussion

### 4.1 Data Description

Table 3 presents the summary statistics of all the study variables. Summary statistics provide an overall picture of the data, including the mean, standard deviation, and minimum and maximum values of the variables for the sampled Chinese firms. The dependent variable DPR has a mean value of 0.227, which means that Chinese firms pay, on average, 22.7% of their earnings in dividends. The average value of DPR is closer to 0.268 (26.8%), as documented by Ref. 58 for a sample of Chinese listed firms. Although this value of the payout ratio falls within the range (20%-30%) fixed by the CSRC, it is quite low. The reason behind This is because minority shareholders’ rights in China are not strong enough to pressure firms to pay dividends. The average CGI score is 0.558, ranging from a low score of 0.221 to a high score of 0.814, which means that, on average, sample firms meet half of the governance standards. The average CSR score of Chinese listed firms is 25.68, which is quite low. The mean value of firm size is 22.118 (similar to 21.60 reported by Ref. 119 and ROA is 0.052, which means, on average, our sampled firms are relatively large and less profitable. The sampled firms have an average debt ratio of 44.64%, indicating their dependency on the debt market, as they rely on the debt market for almost 44.64% of their total assets. The mean values of MTB, RETA, and R&D are 3.590, 0.137, and 0.003, respectively.

**Table 3.** Descriptive Statistics.

Variables	Obs.	Mean	Std. Dev.	Min	Max
DPR	15,799	0.227	0.230	0.000	0.796
CGI	15,799	0.558	0.084	0.221	0.814
CSR	15,799	25.683	18.441	-18.470	90.870
SZ	15,799	22.118	1.180	20.265	24.561
ROA	15,799	0.052	0.044	-0.039	0.147
MTB	15,799	3.590	2.384	1.083	10.277
LEV	15,799	0.446	0.210	0.104	0.817
RETA	15,799	0.137	0.143	-0.239	0.384
R&D	15,799	0.003	0.011	0.000	0.277

Note: For abbreviations and detail of variables, see Table 2.

### 4.2 Correlation matrix

The results of the correlation analysis are presented in Table 4. The correlation matrix provides useful information on the degree and nature of the associations among the variables under study. The absence of multicollinearity is an important assumption of multiple regression models, and according to Ref. 120, multicollinearity exists if the correlation among variables exceeds the cutoff point of 0.80 (80%). The results indicate that the correlation among variables ranges from -0.006 (between leverage and CSR) to 0.528 (between RETA and ROA), showing low-to-moderate levels of correlation. The highest value of correlation is 0.528 (52.8%) between RETA and ROA, which falls below the cutoff point; hence, there is no multicollinearity problem in our model. Moreover, the correlation coefficients of CGI and CSR with DPR were both significant and negative. The coefficient of the correlation between CSR and CG appears to be significantly positive.

**Table 4.** Correlation Matrix.

	DPR	CG	CSR	SZ	ROA	MTB	LEV	RETA	R&D
DPR	1								
CGI	-0.017 *	1							
CSR	-0.264 ***	0.021 **	1						
SZ	0.026 ***	0.064 ***	0.306 ***	1					
ROA	0.059 ***	-0.054 ***	0.095 ***	-0.018 *	1				
MTB	-0.187 ***	0.077 ***	-0.208 ***	-0.345 ***	-0.041 ***	1			
LEV	-0.220 ***	0.021 **	-0.006	0.475 ***	-0.100 ***	-0.038 ***	1		
RETA	0.133 ***	-0.002	0.115 ***	0.051 ***	0.528 ***	-0.157 ***	-0.165 ***	1	
R&D	-0.043 ***	0.029 ***	-0.075 ***	-0.138 ***	-0.006	0.196 ***	-0.090 ***	-0.016 *	1

Note: N=15,799.

Abbreviations and details of the variables are given in Table 2.

* p<0.05.

** p<0.01.

*** p<0.001.

### 4.3 Multivariate analysis

To investigate the mediating role of CSR in the link between dividend policy and corporate governance, we use the mediation approach suggested by Baron and Kenny. ^118^ The results of the mediation analysis are sequentially presented in Table 5.

**Table 5.** Mediation Impact of CSR on Corporate Governance-Dividend Policy Relationship.

Variables	Model I (DPR)		Model II (CSR)		Model III (DPR)		Model IV (DPR)	
	Coeff.	p value	Coeff.	p value	Coeff.	p value	Coeff.	p value
CGI	-0.030	0.029 *	9.001	0.000 ***			-0.011	0.520
CSR					-0.002	0.000 ***	-0.002	0.000 ***
SZ	-0.008	0.000 ***	0.782	0.000 ***	-0.010	0.000 ***	-0.010	0.000 ***
ROA	-0.007	0.604	0.031	0.788	-0.009	0.565	-0.008	0.571
MTB	-0.004	0.001 **	-0.010	0.018 *	-0.003	0.000 ***	-0.005	0.003 **
LEV	-0.005	0.042 *	-0.317	0.044 *	-0.004	0.000 ***	-0.004	0.000 ***
RETA	0.009	0.797	0.136	0.016 *	0.008	0.908	0.008	0.909
R&D	-0.557	0.001 **	-38.377	0.002 **	-0.482	0.003 **	-0.478	0.003 **
Const.	0.438	0.000 ***	13.730	0.000 ***	0.407	0.000 ***	0.410	0.000 ***
R ^2^	0.141		0.165		0.152		0.152	
F statistic (Prob>F)	5.90 (0.000)		14.51 (0.000)		53.01 (0.000)		46.43 (0.000)	

Note: N=15,799.

Abbreviations and details of the variables, see Table 2.

* p <0.05.

** p<0.01.

*** p<0.001.

In Table 5, Model I shows the results for the first condition of mediation, in which the independent variable CGI had a significant impact on the dependent variable DPR in the absence of the mediator variable. Model II shows the results for the second condition of mediation, revealing that the independent variable CGI significantly affects the mediator variable CSR. Model III shows the results for the third condition of mediation, in which the mediator variable significantly affects the dependent variable DPR. Finally, Model IV shows the results for the fourth condition of mediation, in which the effect of the independent variable on the dependent variable decreases and becomes insignificant after the inclusion of the mediator in the model. This is observed from the beta coefficients and p-values of (-.0300364/0.029 and -.0114956/0.520) contained in model I (DPR) and model IV (DPR), respectively.

In model I, our independent variable CG Index is found to have a significant impact on the dividend payout ratio, which confirms the first condition of mediation. The coefficient of the CG index is significantly negative (-.0300364, p<0.05), supporting H1, which states that firms with strong CG practices tend to pay low dividends. The results of Model I provide evidence in favor of the substitution hypothesis of agency theory that dividends act as a substitute for weak corporate governance. In Chinese firms with weak governance, minority shareholders are likely to face strong expropriation from majority shareholders, which results in agency conflicts between them and, hence, the associated agency cost. Dividend policies can help mitigate agency costs. ^121^ Moreover, in firms with poor investor protection, a dividend policy is important for building a strong image by maintaining good relationships with investors. ^25, 100^ Therefore, weak CG firms tend to pay high dividends, and hence, in the weak shareholder rights scenario, high dividends act as substitutes for weak governance. ^42^ However, firms with strong governance are less exposed to these expropriations and experience low agency costs; therefore, they do not need to pay high dividends, indicating an inverse link between dividend policies and corporate governance. Our results are consistent with the findings of previous studies supporting the substitution effect of dividends that firms with high-quality governance tend to pay less dividends (see eg. Refs., 42, 45– 48, 51). Among the control variables, firm size, MTB, leverage, and R&D are significantly and negatively related to the dividend payout ratio (see e.g. Refs. 36, 110) while ROA showed an insignificant negative relationship with the dividend payout ratio, consistent with the findings of Refs. 35 and 50.

In Model II, the coefficient of the CG index is highly significant and positive (9.000815, p<0.001), indicating that firms with good CG tend to make more CSR investments. These results are in accordance with stakeholder theory, which states that apart from shareholders, the consideration of the interests of other stakeholders (e.g., environment, supplier and customers, community, and employees) is critical for corporate success. ^122^ Moreover, our results are in line with the conflict resolution hypothesis, which suggests that firms with effective governance tend to be more socially responsible for resolving conflicts of interest among stakeholders because different stakeholders may have different objectives. ^92, 123^ If these conflicts remain unresolved, they may degrade firm performance. Therefore, in firms with effective corporate governance, managers are more likely to engage in CSR activities to resolve conflicts and maximize shareholder wealth. ^124, 125^

Our results for Model II are consistent with those of Refs. 67, 93 and 95. The significant impact of the CG index on the mediator variable CSR confirms the second condition of mediation. Among the control variables, size is significantly and positively related to dividend payout ratio, while MTB, leverage, and R&D are significantly and negatively related to dividend payout ratio. ROA and RETA remain insignificant.

Model III shows that CSR has a significant negative impact on the dividend payout ratio (-.0020641, p<0.001), indicating that firms with CSR engagements tend to make fewer dividend payments. The results of model III provide evidence for hypothesis H2a and favor the equity cost of the capital channel, which suggests that firms’ CSR involvement reduces the cost of equity capital because these firms are more likely to hoard or invest cash instead of paying dividends. As socially responsible firms enjoy a good reputation, ^63, 64^ have good relationships with stakeholders and are financially less constrained, ^58, 71^ therefore they may have more incentives to hold or invest cash instead of paying dividends. An alternative explanation for the inverse relationship between CSR and dividends, based on the residual theory of dividends, might be that CSR firms tend to pay fewer dividends because CSR investments are costly, so nothing is left to pay as dividends. ^56, 59^ Findings in Model III confirmed the third condition of mediation.

Finally, Model IV shows the impact of CG on dividend policy in the presence of CSR (the mediation effect). The coefficient of the CG index is negative but insignificant (-0.0114956, p>0.05), whereas the coefficient of CSR is significantly negative (-.0020599, p<0.001). Moreover, the value of the CG index coefficient (-.0114956) in Model IV decreased from its value (-.0300364) in Model I, which means that the causal impact of CG on dividend payout ratio is significantly influenced by the presence of CSR. These findings confirm the fourth condition of mediation and provide evidence in favor of our mediation hypothesis H3. The decrease in the coefficient of the CG index in Model IV indicates that CSR mediates the link between CG and the dividend payout ratio. Our results in Model IV are in favor of our second argument about the mediating role of CSR in corporate governance- dividend policy association, which states that better CG leads to high CSR involvement, which in turn reduces dividend payments because of a firm’s preference to hoard cash or invest it instead of paying dividends; hence, CSR mediates the link between CG and dividend policy.

### 4.4 Robustness tests

**4.4.1 Alternative measure of dividend policy**

To check the robustness of our main results on the mediating role of CSR in corporate governance–the dividend policy link–we used a dividend dummy (DD) as a proxy for dividend policy. ^42, 116^ Our dividend dummy takes the value of 1 if the firm is a dividend payer and zero if the firm does not pay dividends.

Table 6 shows the results of the logistic regression models in which the dividend dummy (DD) was used as a dependent variable. Models I, II, III, and IV provide the results for the first, second, third, and fourth conditions of mediation, respectively. In Model I, the CG index was found to have a significant negative impact (-.0927383, p<0.05) on dividend policy, confirming the first condition of mediation in the main model. The results of Model II indicate that the CG index’s impact on CSR is highly significant and positive (7.688139, p<0.001). In Model III, the coefficient value of CSR is -.0689057, with p<0.001 showing a highly significant negative impact on dividend policy. Model IV indicates that the coefficient of the CG index became insignificant after the inclusion of mediator CSR, again supporting our second argument on the mediating role of CSR in the corporate governance-dividend policy link. Overall, the results of the logistic regression analysis are consistent with our main findings presented in Table 5 and support the mediation effect of CSR on the corporate governance- dividend policy relationship.

**Table 6.** Mediation Impact of CSR on Corporate Governance-Dividend Policy Relationship.

(Logistic Regression Model with Dividend Dummy (DD) as a Dependent Variable)								
Variables	Model I (DD)		Model II (CSR)		Model III (DD)		Model IV (DD)	
	Coeff.	p value	Coeff.	p value	Coeff.	p value	Coeff.	p value
CGI	-0.092	0.023 *	7.688	0.000 ***			-0.046	0.838
CSR					-0.068	0.000 ***	- 0.021	0.000 ***
SZ	-0.412	0.000 ***	1.097	0.000 ***	0.229	0.000 ***	0.358	0.000 ***
ROA	-0.011	0.758	-0.014	0.903	-0.038	0.578	-0.015	0.686
MTB	-0.154	0.000 ***	-0.682	0.000 ***	-0.133	0.000 ***	-0.148	0.000 ***
LEV	-3.288	0.000 ***	-4.912	0.000 ***	-2.802	0.000 ***	-3.287	0.000 ***
RETA	1.989	0.000 ***	6.241	0.000 ***	1.882	0.000 ***	1.990	0.000 ***
R&D	-5.916	0.001 **	-28.906	0.018 *	-3.896	0.032*	-5.451	0.002 **
Const.	-6.428	0.000 ***	9.574	0.004 **	-4.211	0.000 ***	-5.522	0.000 ***
Pseudo R ^2^	0.128		0.188		0.231		0.145	

Note: N=15,799.

Abbreviations and details of the variables, see Table 2.

* p <0.05.

** p<0.01.

*** p<0.001.

**4.4.2 Sobel test**

We conducted a Sobel test of mediation to evaluate the significance of the mediation effect of CSR and confirm our main results on the mediating role of CSR that CSR carries the influence of CG on dividend policy. The Sobel test is used to test the statistical significance of a mediation effect ^126^ and is helpful in determining whether there is a significant reduction in the independent variable’s effect on the dependent variable after the inclusion of the mediator. ^127^ Moreover, the Sobel test is appropriate for large samples, and our sample size is quite large. Therefore, using the Sobel test to confirm the mediation effect is applicable in our study. The results of the Sobel test presented in Table 7 confirm the significance of CSR’s mediation effect on the link between CG and dividend policy. Hence, the results of the Sobel test also provide further support for our main finding that CSR influences CG on dividend policy.

**Table 7.** Mediation Impact of CSR on Corporate Governance-Dividend Policy Relationship.

(Sobel Test Results)					
Inputs		Test Statistics	T. Values	St. Error	p value
***a***	0.197	Sobel test	-5.059***	0.005	0.000
***b***	-0.392	Aroian test	-5.057***	0.005	0.000
***S***_***a***_	0.066	Goodman test	-5.061***	0.005	0.000
***S***_***b***_	0.052				

Note: In Table 7, *a* is the regression coefficient for the link between the mediator and independent variables, and *b* is the regression coefficient for the link between the mediator and dependent variable (in the presence of an independent variable). *S _a_* and *S _b_* are the standard errors of the coefficients *a* and *b,* respectively.

**4.4.3 Endogeneity bias**

In accounting research, endogeneity is a common problem that can arise due to omitted variables, explanatory variables, and other instantaneous consequences. ^128, 129^ Until now, we have explored the one-way effect of CG on dividend policy. Reverse causality is implausible because any change in CG requires shareholder approval, and managers consider the governance structure as predetermined. ^130^ On the other hand, dividends are generally determined at managerial discretion. Hence, the former decision is more important than the latter. Thus, causality is likely to be from CG to dividend policy, as reported in the literature (eg. Refs. 10, 36, 121, 131). However, CG and dividend policy may be endogenously determined, and reverse causality may occur from dividend policy to corporate governance. Therefore, to address possible endogeneity bias/reverse causality, we use the GMM model to re-estimate the main model. The results of the GMM model are presented in Table 8, which indicate that there is no endogeneity bias because the p-values of the Sargan test, Hansen test, and AR 2 are all insignificant. Moreover, the signs and coefficients are similar to those of the main models.

**Table 8.** Mediation Impact of CSR on Corporate Governance-Dividend Policy Relationship.

(GMM Model Results)								
Variables	Model I (DPR)		Model II (CSR)		Model III (DPR)		Model IV (DPR)	
	Coeff.	p value	Coeff.	p value	Coeff.	p value	Coeff.	p value
L1	0.618	0.000 ***	0.375	0.000 ***	0.183	0.000 ***	0.182	0.000 ***
CGI	-0.068	0.013 *	21.499	0.000 ***			-0.043	0.346
CSR					-0.001	0.000 ***	-0.001	0.000 ***
SZ	-0.004	0.021 *	1.300	0.000 ***	-0.008	0.000 ***	-0.008	0.000 ***
ROA	-0.014	0.000 ***	0.438	0.000 ***	-0.018	0.000 ***	-0.020	0.000 ***
MTB	-0.001	0.000 ***	-0.004	0.000 ***	-0.002	0.000 ***	-0.002	0.000 ***
LEV	-0.003	0.023 *	-1.649	0.000 ***	-0.026	0.000 ***	-0.029	0.000 ***
RETA	0.001	0.000 ***	0.105	0.000 ***	0.002	0.000 ***	0.002	0.000 ***
R&D	-0.762	0.000 ***	-101.756	0.000 ***	-1.172	0.000 ***	-1.189	0.000 ***
Const.	0.226	0.000 ***	28.114	0.000 ***	0.371	0.000 ***	0.418	0.000 ***
Wald χ ^2^	1729.79		1205.25		1804.59		1905.99	
Prob	0.000		0.000		0.000		0.000	
AR 1 (Prob)	0.000		0.000		0.000		0.000	
AR 2 (Prob)	0.341		0.214		0.490		0.512	
Sargan test (Prob)	0.789		1.000		0.918		1.000	
Hansen test (Prob)	0.592		0.329		0.298		0.743	

Note: N=15,799.

Abbreviations and details of the variables, see Table 2.

* p <0.05.

** p<0.01.

*** p<0.001.

The omitted variable bias may also cause another type of endogeneity. The link between CG quality and dividend payouts is deemed to be spurious because both can be simultaneously determined by omitted variables. ^10^ To control for simultaneity, we used the lagged values of the predictor variables to re-estimate the main model. The results are presented in Table 9, which are consistent with our main findings in Table 5. Hence, our results are insensitive to endogeneity.

**Table 9.** Mediation Impact of CSR on Corporate Governance-Dividend Policy Relationship.

(Model Estimation Using Lagged Values of Predictor Variables)								
Variables	Model I (DPR)		Model II (CSR)		Model III (DPR)		Model IV (DPR)	
	Coeff.	p value	Coeff.	p value	Coeff.	p value	Coeff.	p value
CGI	-0.045	0.036 *	8.481	0.000 ***			-0.017	0.418
CSR					-0.003	0.000 ***	-0.003	0.000 ***
SZ	-0.005	0.001 **	4.193	0.000 ***	-0.008	0.000 ***	-0.008	0.000 ***
ROA	-0.001	0.037 *	0.236	0.043 *	-0.002	0.016 *	-0.002	0.018 *
MTB	-0.004	0.000 ***	-0.014	0.044 *	-0.003	0.000 ***	-0.003	0.000 ***
LEV	-0.034	0.029 *	-1.247	0.016 *	-0.030	0.048 *	-0.030	0.024 *
RETA	0.001	0.085	0.045	0.036 *	0.003	0.035 *	0.003	0.038 *
R&D	-0.731	0.000 ***	-41.999	0.000 ***	-0.593	0.000 ***	-0.588	0.000 **
Const.	0.145	0.000 ***	61.593	0.000 ***	0.346	0.000 ***	0.354	0.000 ***
R ^2^	0.162		0.175		0.185		0.206	
F statistics (Prob> F)	15.31 (0.000)		190.23 (0.000)		213.49 (0.000)		186.94 (0.000)	

Note: N=15,799.

Abbreviations and details of the variables are given in Table 2.

* p<0.05,

** p<0.01,

*** p<0.001.

## 5. Conclusion

Many studies have examined the causal impact of CG on dividend policies. However, the mechanism by which CG affects dividend policies in the presence of CSR remains unexplored. The key intention of this study was to answer the question of whether CSR mediates CG’s impact on firm dividend policy.

To find the answer, we developed two arguments about the mechanism through which CSR can mediate the CG – dividend policy relationship. According to our first argument, we expect that CSR acts as a mediator because firms with strong CG tend to make more CSR investments to guarantee the interest protection of all stakeholders than shareholders (stakeholder theory), and firms with CSR involvement are anticipated to pay more in dividends (earnings channel). Second, since firms with better governance are more likely to invest in CSR-related activities (stakeholder theory) and because of low equity costs, firms tend to pay low dividends when they are involved in CSR activities, as they prefer to hold cash or invest it instead of dividend payments (cost of equity channel). Hence, we hypothesize that CSR could mediate the link between CG and dividend policies.

We used a sample of Chinese listed firms for the period–2011-2021 (consisting of 15,799 firm-year observations) to identify the mediating impact of CSR by applying Baron and Kenny ^118^ mediation method. Our results indicate that the causal link between CG and dividend policy is mediated by CSR, which implies that firms’ investments in CSR have a significant influence on this causal link. Compared to corporate governance, CSR has a more dominant impact on firms’ dividend policy decisions. Further, the results support our second argument about the CSR mediation role that better-governed firms tend to invest more in CSR to protect all stakeholders. As a result, they prefer to hold or invest cash instead of paying dividends because CSR engagements lower the cost of equity capital. The implication of our findings is that better-governed Chinese firms are more likely to engage in CSR to safeguard their stakeholders, and they do not need to use costly dividends to convey private information on their daily operations to their investors because firms’ CSR activities provide more useful and precise information. Our findings are corroborated by a set of robustness tests, including logistic regression, the Sobel test, and possible endogeneity bias.

## 6. Implications and limitations

Our results show that CSR carries the CG influence on dividend policy decisions has implications for firms, regulators, and investors. For firms, our findings suggest that weak CG can be substituted with high dividend payouts to compensate for poor investor protection and maintain good relationships with investors. For investors, our findings that better-governed firms invest more in CSR suggest that investors should pay close attention to firms’ CSR engagements while making investment decisions because socially responsible firms can provide better protection to investors due to their strong CG structure and as compared to dividends, CSR activities can also be an indicator of meaningful information on firms’ daily activities. For regulators, policymakers are advised to give special consideration to CSR to reduce environmental and social problems and to enhance the related standards to ensure the safety and security of all stakeholders, thereby reducing global accusation and pressure. Firms can use high dividend payouts to compensate for poor investor protection and to maintain good relationships with investors. When making investment decisions, investors are advised to consider socially responsible firms because of their strong CG structure. Finally, policy makers should give special consideration to CSR in order to reduce environmental and social problems and to enhance the related standards to ensure the safety and security of all stakeholders and hence reduce global accusation and pressure.

Since we considered Chinese firms as study samples, our findings that CSR mediates the causal link between CG and dividend policy may or may not be generalizable to other settings. Future studies could test the mediating impact of CSR in the context of both developed and other emerging economies for international comparability.

## Declaration

### Ethical approval statement

Not applicable.

## Data Availability

The data used in this study is upload in repository at https://doi.org/10.6084/m9.figshare.29890247.v1. ^132^ Also, CSR data were collected from the Hexun database. The data on all other variables, including CG and control variables, were collected from the China Stock Market and Accounting Research (CSMAR) database. The original data used in this study are accessible at https://data.csmar.com (CG and financial variables and at http://stockdata.stock.hexun.com/zrbg/Plate.aspx (For CSR score). The dataset has a CC-BY 4.0 license applied.

## Footnotes

^1^
See: Levisohn, B. (2013). Don’t Ignore Dividends When Investing in China. *Emerging Markets Daily,* 19 February. Article available at https://www.barrons.com/articles/BL-231B-1394.
