# Supplementary material for: Revisiting the Governance–Dividend Nexus: The Mediating Role of Corporate Social Responsibility
Source: F1000Res. 2026 Mar 31;14:886. Originally published 2025 Sep 9. [Version 3] doi: 10.12688/f1000research.167892.3 (PMC13062773; doi:10.12688/f1000research.167892.3)
Supplement: Peer review history

# Reviewer report: Reviewer response for version 3

**DOI:** 10.5256/f1000research.197783.r471975

**Published:** 2026-04-09

**Authors:** Zainal Abidin

**Affiliations:** 1 Sekolah Tinggi Ilmu Ekonomi Indonesia Jakarta, East Jakarta, Indonesia

The revised manuscript presents a clear and well-structured analysis of the relationship between corporate governance, dividend policy, and CSR using panel data from Chinese listed firms. The authors have made meaningful improvements compared to the previous version, particularly in clarifying the conceptual positioning of CSR and moderating earlier normative interpretations. The discussion now more appropriately reflects the limitations of CSR as a proxy for ethical or sustainability outcomes, which strengthens the overall credibility of the study.

 Methodologically, the paper remains sound, employing appropriate panel data techniques and presenting results in a transparent manner. The conclusions are now better aligned with the empirical evidence, avoiding overstatement and maintaining a clearer distinction between financial relationships and broader normative implications. The manuscript is also well written and suitable for the journal’s readership.

 Overall, the key concerns raised in the previous review have been adequately addressed. The paper is now scientifically sound, and any remaining issues are minor and relate mainly to clarity and conciseness rather than substantive weaknesses. I therefore consider the manuscript suitable for indexing in its current form.

Is the work clearly and accurately presented and does it cite the current literature?

Partly

If applicable, is the statistical analysis and its interpretation appropriate?

Partly

Are all the source data underlying the results available to ensure full reproducibility?

Partly

Is the study design appropriate and is the work technically sound?

Partly

Are the conclusions drawn adequately supported by the results?

Partly

Are sufficient details of methods and analysis provided to allow replication by others?

Partly

Reviewer Expertise:

CSR and sustainability

I confirm that I have read this submission and believe that I have an appropriate level of expertise to confirm that it is of an acceptable scientific standard.

# Reviewer report: Reviewer response for version 2

**DOI:** 10.5256/f1000research.192747.r454073

**Published:** 2026-02-11

**Authors:** Zainal Abidin

**Affiliations:** 1 Sekolah Tinggi Ilmu Ekonomi Indonesia Jakarta, East Jakarta, Indonesia

**Journal Scope and Suitability**

 Based on the aims and scope of the journal, this manuscript is **generally suitable** for the journal’s readership. The topic—linking corporate governance, dividend policy, and CSR—is relevant to scholars interested in corporate finance, governance, and sustainability-related issues, particularly in emerging market contexts. The empirical focus and quantitative approach are consistent with the type of research typically published by the journal.

**2. Summary of the Paper and Overall Assessment**

 This paper examines the relationship between corporate governance and dividend policy, proposing corporate social responsibility (CSR) as a mediating mechanism. Using panel data from Chinese listed firms over the period 2012–2021, the authors construct a composite corporate governance index and employ CSR scores from the Hexun database. Fixed-effects panel regressions are used to test both direct and mediated relationships.

 The main findings suggest that stronger corporate governance is associated with lower dividend payouts, that governance positively influences CSR engagement, and that CSR partially mediates the governance–dividend relationship. Overall, the study offers a solid empirical analysis using a large dataset. However, several conceptual and interpretive issues limit the manuscript’s scientific robustness in its current form.

**3. Originality, Relevance, and Contribution**

 The manuscript makes an **incremental contribution** by explicitly modeling CSR as a mediating mechanism between governance and dividend policy in an emerging market setting. While the topic is relevant and of interest to the journal’s readership, the originality lies more in the empirical configuration than in theoretical development.

 The contribution would be stronger if the authors more clearly positioned their work within broader debates on CSR and sustainability governance, rather than relying predominantly on an instrumental, agency-based interpretation of CSR.

**4. Methodology and Validity**

 The empirical methodology is generally appropriate. The use of panel data, fixed-effects estimation, and standard control variables is consistent with accepted practice in this field. Tables and results are clearly presented, and the analysis appears internally consistent.

 That said, the study implicitly assumes that higher CSR scores reflect stronger ethical or sustainability-oriented governance. This assumption is not sufficiently discussed or justified, particularly given that CSR scores may capture disclosure intensity or legitimacy-seeking behavior rather than substantive social or environmental impact.

**Suggestion:**

 The authors should more explicitly acknowledge the limitations of CSR scores as proxies for sustainability or ethical performance and clarify that the analysis primarily captures financial governance behavior.

**5. Interpretation of Results and Conclusions**

 The interpretation of the empirical results occasionally goes beyond what the data can directly support. In particular, the discussion suggests that firms are prioritizing social responsibility over shareholder payouts, which introduces a normative interpretation that is not directly evidenced by the analysis.

**Suggestion:**

 The conclusions should be more cautiously framed, clearly distinguishing between observed financial allocation patterns and broader ethical or sustainability implications.

**6. Presentation, Clarity, and Length**

 The manuscript is generally well written and clearly structured. The length is appropriate for the subject matter, and the tables and figures are helpful. Minor improvements in clarity could be achieved by tightening parts of the discussion section, particularly where interpretations become speculative.

 I did not identify major linguistic issues, although a light stylistic review could further improve clarity in some sections.

**7. Aspects Outside My Expertise**

 I do not assess the technical construction of the proprietary CSR scoring methodology itself, as this lies outside my direct expertise. My comments focus on conceptual interpretation rather than the internal mechanics of the CSR index.

Is the work clearly and accurately presented and does it cite the current literature?

Partly

If applicable, is the statistical analysis and its interpretation appropriate?

Partly

Are all the source data underlying the results available to ensure full reproducibility?

Partly

Is the study design appropriate and is the work technically sound?

Partly

Are the conclusions drawn adequately supported by the results?

Partly

Are sufficient details of methods and analysis provided to allow replication by others?

Partly

Reviewer Expertise:

CSR and sustainability

I confirm that I have read this submission and believe that I have an appropriate level of expertise to confirm that it is of an acceptable scientific standard, however I have significant reservations, as outlined above.

## References

[1] : Corporate Social Responsibility Reporting as Substantive and Symbolic Behavior: A Multilevel Theoretical Analysis. *Business and Society Review* .2016;121(2) : 10.1111/basr.12089 297-327 10.1111/basr.12089

[2] : CSR beyond symbolism: The importance of substantive attributions for employee CSR engagement. *Journal of Cleaner Production* .2024;436: 10.1016/j.jclepro.2023.140440 10.1016/j.jclepro.2023.140440

[3] : Meta-analyses on Corporate Social Responsibility (CSR): a literature review. *Management Review Quarterly* .2022;72(3) : 10.1007/s11301-021-00211-2 627-675 10.1007/s11301-021-00211-2

[4] : Corporate social responsibility and dividend policy. *Research in International Business and Finance* .2019;47: 10.1016/j.ribaf.2018.07.005 114-138 10.1016/j.ribaf.2018.07.005

[5] : The relationship between dividend payout and corporate social responsibility: The moderating effect of shareholder friendliness and board monitoring. *Journal of Cleaner Production* .2023;394: 10.1016/j.jclepro.2023.136297 10.1016/j.jclepro.2023.136297

[6] : Mind the gap: The interplay between external and internal actions in the case of corporate social responsibility. *Strategic Management Journal* .2016;37(13) : 10.1002/smj.2464 2569-2588 10.1002/smj.2464

# Response

**Published:** 2026-03-15

**Authors:** Hussaini Bala

**Affiliations:** Accounting, Tishk International University, Erbil, Iraq

**Responses to Reviewers**

**Submission ID: 167892 #TrackingId:24443388**

 Dear Respected Reviewers  

 Thank you so much for the time you have put into examining this manuscript and doing a constructive review. Your comments have helped us correct all the noted errors and bring clarity to this work. We truly appreciate it.

 For clarity and ease of understanding, the revisions that we have made are all highlighted using RED text in the revised paper. Also, line numbers are included in the margin of the revised paper to easily locate the revisions made. Based on your comments, the following improvements have been made with supporting references. 

 Thank you for your time and consideration.

 Reviewer 1# Comments:

**S/No**

**Reviewers Comments**

**Action/response from Authors**

 1

 The main findings suggest that stronger corporate governance is associated with lower dividend payouts, that governance positively influences CSR engagement, and that CSR partially mediates the governance–dividend relationship. Overall, the study offers a solid empirical analysis using a large dataset. However, several conceptual and interpretive issues limit the manuscript’s scientific robustness in its current form.

**Response:** We would like to sincerely thank the reviewer for his constructive evaluation and acknowledgment of the empirical contribution of the manuscript. We appreciate the positive acknowledgment that the manuscript provides a solid empirical analysis based on a large dataset and that it identifies important relationships among corporate governance, CSR, and dividend policy.

 With respect to the concern of the reviewer about the manuscript’s conceptual and interpretive aspects, we have carefully reviewed the manuscript and made some improvements to strengthen its scientific robustness. First, the manuscript’s conceptual framework has been further clarified to better articulate the theoretical mechanisms between corporate governance and dividend policy through CSR engagement. In particular, the manuscript’s interpretation of the theoretical framework now more clearly integrates agency theory and stakeholder theory to better explain how good corporate governance might encourage companies to invest in sustainability initiatives instead of dividend payments.

 Second, we have expanded the interpretation of the empirical findings to better explain the identified relationship between corporate governance and dividend policy through CSR engagement. In particular, the manuscript’s interpretation of the empirical findings now more clearly discusses why good corporate governance might be related to lower dividend payments and how CSR investments might be an alternative channel for value creation and long-term stakeholder engagement.

 See the revised introduction, literature review, methodology, results and discussion, and conclusion and implication sections as highlighted in red.

 2

 The manuscript makes an incremental contribution by explicitly modeling CSR as a mediating mechanism between governance and dividend policy in an emerging market setting. While the topic is relevant and of interest to the journal’s readership, the originality lies more in the empirical configuration than in theoretical development.

 The contribution would be stronger if the authors more clearly positioned their work within broader debates on CSR and sustainability governance, rather than relying predominantly on an instrumental, agency-based interpretation of CSR.

**Response: **We appreciate the reviewer for his helpful suggestion. In response to this, we have revised the contribution in the introduction to more clearly position the study within broader debates on CSR and sustainability governance.

 See the revised introduction as highlighted in red.

 3

**Methodology and Validity**

 The empirical methodology is generally appropriate. The use of panel data, fixed-effects estimation, and standard control variables is consistent with accepted practice in this field. Tables and results are clearly presented, and the analysis appears internally consistent.

 That said, the study implicitly assumes that higher CSR scores reflect stronger ethical or sustainability-oriented governance. This assumption is not sufficiently discussed or justified, particularly given that CSR scores may capture disclosure intensity or legitimacy-seeking behavior rather than substantive social or environmental impact.

**Suggestion:**

 The authors should more explicitly acknowledge the limitations of CSR scores as proxies for sustainability or ethical performance and clarify that the analysis primarily captures financial governance behavior.

**Response: **We thank the reviewer for this valuable comments. In response to this suggestion, we have added a clarification in the methodology admitting the limitations of CSR scores as proxies for substantive sustainability or ethical performance.

 See the revised methodology as highlighted in red.

 4

**Interpretation of Results and Conclusions**

 The interpretation of the empirical results occasionally goes beyond what the data can directly support. In particular, the discussion suggests that firms are prioritizing social responsibility over shareholder payouts, which introduces a normative interpretation that is not directly evidenced by the analysis.

**Suggestion:**

 The conclusions should be more cautiously framed, clearly distinguishing between observed financial allocation patterns and broader ethical or sustainability implications.

**Response: **We thank reviewer for this valuable comment. In response to the comment, we have now revised the conclusions and limitation to clearly differentiate observed financial patterns from wider ethical or sustainability interpretations

 See the revised conclusion and implications as highlighted in red.

 5

 The manuscript is generally well written and clearly structured. The length is appropriate for the subject matter, and the tables and figures are helpful. Minor improvements in clarity could be achieved by tightening parts of the discussion section, particularly where interpretations become speculative.

 I did not identify major linguistic issues, although a light stylistic review could further improve clarity in some sections.

**Response: **We appreciate the reviewer for the positive comments. We  have now tightened the introduction, literature review, methodology, results and discussion, conclusion and implication sections as highlighted in red.

 See the revised introduction, literature review, methodology,  results and discussion, conclusion and implication sections as highlighted in red.

 We believe the revisions we have made significantly enhance the clarity and depth of our analysis. Your invaluable input has greatly strengthened our work, and we are deeply grateful for your assistance.

 Thank you for your time and help.

 Best Regards,

 The Authors

# Reviewer report: Reviewer response for version 1

**DOI:** 10.5256/f1000research.185036.r413711

**Published:** 2025-10-06

**Authors:** Noor Afza Amran

**Affiliations:** 1 Universiti Utara Malaysia, Sintok, Kedah, Malaysia

The abstract has been discussed clearly and includes all the necessary items to be included in an Abstract.

 The problem statement clearly highlights the role of CSR in mediating CG and dividend payout.  This study adopts agency and stakeholder theories for application.

 The LR section is comprehensive and up-to-date. 

 For the methodology section, the years of study from 2012 to 2021 are adequate. Using a secondary data approach.

 Sample of the 2012 China Securities Regulatory Commission (CSRC). 

 For the CGI Index, we need to explain whether the Index is constructed by researchers or adopted from another source. If it is adopted/adapted from another source, please acknowledge in this section. 

 If you developed the CGI, then you did not mention how you conducted validity and reliability tests of your construct. The validation of the construct needs to be explained clearly.

 Results and discussion are adequate.

Is the work clearly and accurately presented and does it cite the current literature?

Yes

If applicable, is the statistical analysis and its interpretation appropriate?

Yes

Are all the source data underlying the results available to ensure full reproducibility?

Yes

Is the study design appropriate and is the work technically sound?

Yes

Are the conclusions drawn adequately supported by the results?

Yes

Are sufficient details of methods and analysis provided to allow replication by others?

Partly

Reviewer Expertise:

Corporate governance, Auditing, Sustainability, Financial Reporting

I confirm that I have read this submission and believe that I have an appropriate level of expertise to confirm that it is of an acceptable scientific standard.
